# Extracellular Vesicle-DNA: The Next Liquid Biopsy Biomarker for Early Cancer Diagnosis?

**DOI:** 10.3390/cancers15051456

**Published:** 2023-02-24

**Authors:** Irène Tatischeff

**Affiliations:** Honorary CNRS and UPMC Research Director, Founder of RevInterCell, a Scientific Consulting Service, 91400 Orsay, France; irene.tatischeff@upmc.fr

**Keywords:** extracellular vesicles (EVs), exosomes (EXs), liquid biopsy (LB), EV-associated DNA (EV-DNA), cancer diagnosis

## Abstract

**Simple Summary:**

Each human cancer is a specific disease, but all cancers share the necessity of an early diagnosis for providing the optimal outcome for the patient. Liquid biopsy in blood, as a substitute to invasive tissue biopsies, brought the first important breakthrough for cancer diagnosis. The race for efficient cancer biomarkers was first focused on the few circulating tumor cells released in the bloodstream, then on circulating cell-free tumor DNAs in plasma and serum. The last decade’s discovery of the ubiquitous cell-derived extracellular vesicles (EVs) brought a new “treasure chest” for the worldwide search of cancer biomarkers among the many tumor EVs-associated biological components. The aim of this review is to follow the different steps—mostly in vitro and preclinical liquid biopsies—which focused the current interest on tumor EVs-associated DNA as a promising cancer biomarker that still has many challenges yet to be solved before reaching the clinic.

**Abstract:**

After a short introduction about the history of liquid biopsy, aimed to noninvasively replace the common tissue biopsy as a help for cancer diagnosis, this review is focused on extracellular vesicles (EVs), as the main third component, which is now coming into the light of liquid biopsy. Cell-derived EV release is a recently discovered general cellular property, and EVs harbor many cellular components reflecting their cell of origin. This is also the case for tumoral cells, and their cargoes might therefore be a “treasure chest” for cancer biomarkers. This has been extensively explored for a decade, but the EV-DNA content escaped this worldwide query until recently. The aim of this review is to gather the pilot studies focused on the DNA content of circulating cell-derived EVs, and the following five years of studies about the circulating tumor EV-DNA. The recent preclinical studies about the circulating tEV-derived gDNA as a potential cancer biomarker developed into a puzzling controversy about the presence of DNA into exosomes, coupled with an increased unexpected non vesicular complexity of the extracellular environment. This is discussed in the present review, together with the challenges that need to be solved before any efficient clinical transfer of EV-DNA as a quite promising cancer diagnosis biomarker.

## 1. Introduction

Cancer is a major burden on humanity, as recapitulated by Globocan, the Global Cancer Statistics 2020, concerning 36 cancers, with regard to their respective incidence and mortality in men and women from 185 countries worldwide (https://acsjournals.onlinelibrary.wiley.com/doi/10.3322/caac.21660, accessed on 31 October 2021). Human cancer is still a mysterious multiform disease, and each organ-specific cancer has to be considered as a unique disease, with some common hallmarks. They also share in common the necessity of an early diagnosis for an optimal outcome for the patient. Besides the many sophisticated technologies now available for asserting a cancer diagnosis, liquid biopsy, first in blood (serum, plasma) and now in many other body fluids (urine, cerebrospinal fluid, saliva), has brought the hope of an efficient cancer signature for significantly helping an early diagnosis. Many components released from the tumor cell machinery during life and death might be candidates for being noninvasive cancer “whistleblowers”, which explain the already long-lasting query about the most promising liquid biopsy biomarkers. Liquid biopsy in blood already has a long history as a promising substitute for tissue biopsy for cancer diagnosis. Cancer biomarkers were first focused on rare circulating tumor cells (CTCs), followed by cell-free tumor DNAs (cf-tDNAs). Recently, circulating tumor extracellular vesicles (cir-tEVs) became the third most interesting resource for cancer liquid biopsy [1,2]. The high EVs heterogeneity has been classified into three main EV categories, according to their size, biogenesis, composition and biological properties [3,4]: apoptotic bodies (ABs) (50 nm–5 µm in diameter), microvesicles (MVs) (100 nm–1 µm) and exosomes (EXs) (30 nm–150 nm). Due to the lack of specific vesicle biomarkers and the EVs overlapping size properties, it is presently difficult to efficiently discriminate the different EVs; therefore, they currently share the generic name of extracellular vesicles (EVs) [5]. The release of different types of extracellular vesicles (EVs) is recognized as a new important common cell property, extending each cell influence well beyond its plasma membrane. For about a decade, EVs have been recognized as important messengers of intercellular communication, and nowadays, their major biological functions in human health and disease are highly investigated. With regard to their recent involvement as circulating EVs (cirEVs) in many body fluids for diagnosis of human diseases including cancers, the smallest vesicles, i.e., mainly exosomes, are the most considered. As recently reviewed [1,2], an increasing worldwide search is focused on finding the most relevant biomarkers to achieve early diagnosis of different human cancers among the many macromolecular components, which are specifically carried inside the rich cargoes of the numerous circulating tumor EVs (cir-tEVs). Although cf-tDNAs was the second important resource for cancer liquid biopsy, EV-DNA remained long ignored as a tumoral biomarker.

The aim of the present review is to point out the recent studies which shed light on the potential capacity of cir-tEV-DNA as a new, interesting biomarker candidate for early diagnosis and prognosis of human cancers. The current knowledge evolution about the composition of the extracellular medium will also be discussed, as well as the challenges to solve before any usable routine clinical transfer.

## 2. Pilot Studies Focused on the DNA Content of Circulating Cell-Derived EVs (2011–2016)

After the mere observation that tumor cells release more EVs (tEVs) than their normal cell counterparts (nEVs), it was obviously interesting to check the comparative cargo composition of tEVs and nEVs. This EV cargo comparison was first focused on EV proteins, then on EV-RNAS (coding messenger RNAs (mRNAs), and mainly noncoding RNAs (microRNAs (miRNAs), long noncoding RNAs (lncRNAs), and circular RNAs (cirRNAs)). At first, small EVs were not supposed to harbor DNAs, which was only assumed as a known property of apoptotic bodies. However, in 2011, Balaj et al. [6] asserted that tumor cells release an abundance of microvesicles containing a selected set of proteins and RNAs. However, they also carry DNA, which reflects the genetic status of the tumor, including a significant sequence amplification of the *c-Myc* oncogene for three medulloblastoma cell lines compared with normal fibroblasts and other tumor cell types. ExoDNA appeared to be primarily single stranded (ssDNA). Tumor microvesicles contain genetic information available for horizontal gene transfer and provide a potential source of tumor biomarkers. In 2012, Waldenström et al. [7], after having previously revealed that human prostasomes contain chromosomal DNA, successfully searched DNA in microvesicles/exosomes derived from a murine cardiomyocite cell line; they also showed that these EVs, containing DNA/RNA, could transfer chromosomal DNA sequences to the cytosol or nuclei of target fibroblasts. These two pioneering works on MVs initiated the interest in EV-DNA.

In 2013, six years after the noticeable observation of Valadi et al. [8], claiming that “exosome-mediated transfer of mRNAs and microRNAs is a novel mechanism of genetic exchange between cells”, Cai et al. [9] showed that “extracellular vesicle-mediated transfer of donor genomic DNA to recipient cells is a novel mechanism for genetic influence between cells”. They first examined the existence of genomic DNA (gDNA) in EVs derived from human plasma and from vascular smooth muscle cells (VSMCs) in culture. They found at least 16,434 gene fragments in the human plasma, ranging in size from 1 to 20 kilobases (kb), but mostly around 17 kb. They showed with VSMCs that apoptosis was not the source of EV-DNA. Moreover, they observed that DNA was present only inside the thoroughly washed EVs and that EVs contain double-stranded DNA (dsDNA). Then, they investigated the function of transferable DNA in the recipient cells. To determine the pathophysiological significance of EV-gDNA transfer into cells, they further examined the transfer of BCL/ABL hybrid gene in EVs from K562 cells to normal human neutrophils isolated from human peripheral blood. They found that the numerous gDNA fragments in EVs are transportable between the same or different types of cells and increase the gDNA-coding mRNA and protein expressions in the recipient cells. This immediately boosted the interest of circulating EV-DNA as a new cancer biomarker in liquid biopsy.

In 2014, independently of the three pilot studies detailed above, Kahlert et al. [10] investigated whether exosomes from two pancreatic cancer cell lines and serum from (a few) patients with pancreatic ductal adenocarcinoma (PDAC) contain gDNA. They provided evidence that exosomes contain >10 kb fragments of ds-gDNA spanning all chromosomes. They showed that the known specific KRAS and p53 DNA mutations found in the pancreatic tumor cells were recovered in the serum exosomes of patients with pancreatic cancer. Therefore, serum exosomes might be used to determine gDNA mutations for cancer prediction. Moreover, their data suggested that the majority of circulating DNAs from the serum samples may come from inside the exosomes and are not present as free-floating circulating DNA. This important preliminary study opened the way to a preclinical liquid biopsy study, involving a larger number of PDAC patients, compared to the appropriate healthy controls.

At the same time, Thakur et al. [11] used a quite interesting approach to evidence, for the first time, dsDNA in exosomes derived from two human cancer cell lines (myeloid leukemia K562 and colorectal carcinoma HCT116) and one murine melanoma cell model. They extracted DNA from exosomes either intact or pretreated with DNAse. However, instead of using the nonspecific DNAse I, they used either S1 nuclease, specific for ssDNA, or shrimp-dsDNAse, specific for dsDNA. Thus, they convincingly demonstrated that the majority of exosome-dsDNA with a size greater than 2.5 kb is associated with the outer membrane, whereas internal exosome-dsDNA depicts a size between 100 bp and 2.5 kb. This first observation of exosome-associated dsDNA was confirmed by atomic force microscopy (AFM) and extended a broad panel of human tumor cell lines to the analysis. A predominant dsDNA form of internal exoDNA was detected in all exosomes, but with lower amounts in most pancreatic and lung cancer cell lines. It is noticeable that exosomes from two normal fibroblast stromal cell lines exhibited about 20-fold less exoDNA than the one isolated from tumor cells. Furthermore, exosomes derived from murine B16-F10 melanoma revealed that only a (10%) subset of exosomes contained DNA, suggesting a specific targeting of DNA into exosomes. Another very important point of this work is that exoDNA represents the entire genome and might then mirror the tumor state. Focusing on a major modification of nuclear DNA, i.e., the methylation of 5’-cytosine, exoDNA was found methylated to a similar level to gDNA. ExoDNAwas also tested for some cancer-specific mutations such as the BRAF (V600E) mutation, present in 50% of malignant melanoma. They detected the mutant alleles in exoDNA of all cell lines containing the mutation and only the wild type (WT) in exoDNA originating from the cell lines with non mutated BRAF. The same search was performed with the epidermal growth factor receptor (EGFR), which is mutated in several cancers, including non small cell lung cancer (NSCLC), and respective EGFR mutations were also detected in 100% of exoDNA isolated from the NSCLC cell lines, harboring EGFR mutations. Thus, Thakur et al. [11] showed that double-stranded DNA in exosomes reflects the mutational status of parental tumor cells, illustrating its significant translational potential as a novel circulating biomarker candidate in cancer detection.

Lee et al. [12] used (RAT-1), an immortalized nontumorigenic rat intestinal epithelial cell line (IEC-18) and its tumoral derivative (RAS-3) transfected with the V12 mutant c-H-ras human oncogene. By whole genome sequencing (WGS), these EVs, containing chromatin-associated dsDNA large fragments (777 bp, 2200 bp), were shown to cover the entire rat genome, including the full-length H-ras oncogene (3308 bp). Moreover, these EVs could transfer this oncogene to nontumorigenic cells and induce their increased proliferation.

After evidencing gDNA inside microvesicles/exosomes [6,7,9,10,11,12], the presence of DNA was also questioned in other EVs [13,14]. Shelke et al. [13] claimed that the EV-DNA released by human mast cells is mostly associated with the outside of EVs and cause their aggregation. Fisher et al. [14] showed that EVs (50–150 nm in size) released from human bone marrow-derived mesenchymal stromal cells (BM-hMSC) also carry high-molecular DNA. This DNA, which is not derived from apoptotic or necrotic cells, was mainly associated with the outer EV membrane and, to a smaller degree, inside the EVs. The DNA isolated from EVs was not organized in nucleosomes. The EV-gDNA amount was sufficient for next-generation sequencing (NGS) and virtually covered the complete human genome. After transducing a plant-DNA into BM-hMSCs, the released EVs were tagged with the *Arabidopsis thaliana*-DNA (*A.t.*-DNA) and able to rarely perform the (*A.t.*-DNA) EV-mediated transfer to naïve BM-hMSCs. As previously observed with rat cells [12], this is a confirmation of the EV-mediated horizontal DNA gene transfer to recipient cells as a new important EV biological function. In 2016, Kalluri and Lebleu summarized the discovery of double-stranded genomic DNA in circulating exosomes [15], focusing on studies related to the origin of gDNA in exosomes and its utility in cancer diagnosis and disease monitoring. Lastly, Jin et al. [16] proved that EVs extracted from serum are stable under different storage conditions (at 4 °C for 24 h, 72 h, 168 h; at room temperature for 6 h, 12 h, 24 h, 48 h; and after one-time-, three-time-, five-time-repeated freeze–thaw cycles). DNA in serum EVs is also stable under different storage conditions. Serum DNA is mainly present in exosomes, and EVs-DNA stayed stable for 1 week at 4 °C, 1 day at room temperature, and after fewer than three-time-repeated freeze–thaw cycles. The observed stability of serum EVs and EVs-DNA is the premise for using cirEVs for the search of new potential genetic DNA biomarkers for cancer diagnostics. A summary of these precursor studies on the DNA content of cell-released EVs is given in Table 1.

## 3. Following Studies about the Circulating Tumor EV-DNA (2014–2019)

First, Lazaro-Ibanez et al. [17] showed different gDNA fragments in the subpopulations of EVs (Abs, MVs, and EXs) with prostate cancer (PCa) cell lines (LNCaP, PC-3, and R92a/hTERT) in vitro. Derived from morphologically heterogeneous cancer cells, their respective MVs and EXs had comparable sizes and concentrations (1.36–2.52 × 10^8^ particles/mL per million cells) for MVs (n = 16) and (0.56–1.93 × 10^8^ particles/mL per million cells) for EXs (n = 16). However, for each of the three cell lines, the MVs’ total protein content (6 µg protein/10^6^ cells) was about twice that of EXs (3.2 µg protein/10^6^ cells). Besides very rare MLH1 mutations in prostate cancer (PCa), TP53 and PTEN were the only significantly mutated genes in both localized PCa and castration-resistant (CRPC) tumors. The number of amplified gDNA fragments of MLH1 (108 bp), PTEN (225 bp), and TP53 (316 bp) were almost double between MVs and EXs (n = 12), showing that different types of EVs carried different gDNA contents, which suggests a selective gDNA package into the different PCa cell-derived EV subtypes. Moreover, they demonstrated that the EV-derived gDNA fragments from the LNCaP cells had no MLH1 mutation but a frame-shift PTEN mutation and a (C > G) TP53 mutation, showing that EV-gDNA could even harbor specific gDNA mutations of the parent cells. Then, they provided evidence that plasma-derived EVs are more abundant in PCa patients (n = 4) than in healthy donors (n = 4) and that human plasma-derived EVs also carry double-stranded gDNA fragments. However, they did not observe any significant differences in the MVs and EXs or in the total EV population isolated from human plasma samples of PCa patients compared with healthy controls. Moreover, the previously described gDNA mutations for the LNCaP cell-derived EVs were not detected from the small studied cohort of plasma EVs. Thus, the promising in vitro observations are to be confirmed by other extended preclinical studies, before asserting EV-DNA as a valuable biomarker for PCa diagnostics.

After previous isolation from tumor cells with high migratory and invasive abilities of new, unusually large (1 µm in diameter) EVs (L-EVs), also named large oncosomes (LO), Vagner et al. [18] first characterized the DNA in large L-EVs (LO surrogate) and small S-EVs (EX surrogate) from the same PC3 (PCa) or U87 (glioblastoma) cancer cell lines, as well as from plasma of a PCa mouse model. L-EVs emerged as the EV subpopulation containing most of the circulating DNA, which was quantified as a high molecular weight (up to 2 Mb) chromatinized DNA. Then, they isolated L-EVs and S-EVs from human plasma of patients (n = 40) with metastatic castration-resistant PCa (mCRPC). As observed in vitro, and despite a pronounced interpatient variability in the amount of EV DNA, L-EVs contained significantly more DNA than S-EVs, whereas DNA was totally absent from both L-EVs and S-EVs in controls. Moreover, L-EVs isolated from human mCRPC patients contained large-size dsDNA, covering the entire tumor genome, with reported cancer-specific genomic alterations (MYC/PTEN imbalance). It is noticeable that, in line with their in vitro and in vivo results, the ssDNA/dsDNA ratio was 5/1 in three out of four patients and the amount of EV-free DNA was comparable or higher than the amount of DNA in L-EVs in two patients. This points out the necessity for further preclinical studies to shed light on the relationship between disease progression and the composition of the DNA cargo in L-EVs.

Pancreatic cancer, in urgent need of early diagnosis, was also considered under the light of DNA biomarker. Allenson et al. [19] compared exosome-derived DNA (exoDNA) to cfDNA in liquid biopsies of patients with pancreatic ductal adenocarcinoma (PDAC) on 263 individuals, including a discovery cohort of 68 PDAC patients of all stages, 20 PDAC patients with localized tumor after curative resection, and 54 healthy controls. A validation cohort of 39 cancer patients and 82 healthy controls was studied to validate KRAS detection rates in early-stage PDAC patients. *KRAS* mutations were more detectable in exoDNA than in cfDNA. However, mutant *KRAS* was also detected in a substantial minority of healthy samples, which limits its utility as a cancer-screening method. Yang et al. [20] added to the search of *KRAS^G12D^* mutation in serum exosomal DNA, the associated search of *TP53^R273H^* mutation from patients with pancreatic cancer and healthy individuals. The minimal exosomal DNA used for digital PCR analyses was 0.663 ng. A sufficient amount of exosomal DNA for the *KRAS^G12D^* and *TP53^R273H^* mutations search was obtained for 49% (76/156) of patients and 66% (114/171) of healthy serum samples. In 39.6% of the serum samples of PDAC patients (n = 48), the *KRAS^G12D^* mutation was identified, whereas the *TP53^R273H^* mutation appeared in 4.2% of the serum samples, leaving 27 samples without these two specific mutations. With the frequency of the *KRAS^G12D^* mutation being measured as about 40–50% in PDAC tumor tissue, this exosomal DNA study likely captures most of the *KRAS^G12D^* mutation in PDAC patients. This also appears to be the case for the *TP53^R273H^* mutation. Thus, this study showed that exosomal DNA can be used as a substitute for less convenient tissue biopsy to identify mutations using digital PCR. Moreover, whereas *KRAS^G12D^* mutation was detected in 2.6% of a large cohort (n = 114) of healthy individuals, *TP53^R273H^* mutation was never detected in healthy subjects.

On the other hand, in vitro and in vivo studies [21] showed the interest of engineered exosomes (iExosomes) to carry short interfering RNA (siRNA) or short hairpin RNA (shRNA), specific to oncogenic *Kras^G12D^*, for efficiently targeting *KRAS*. Mendt et al. [22] reported a bioreactor-based generation and testing of large-scale production of clinical-grade iExosomes for targeting *KRAS* in pancreatic cancer. These iExosomes were thoroughly tested in vitro with many cell lines and in vivo on several mouse models with pancreatic cancer. These studies confirmed the suppression of oncogenic *Kras* and an increase in the survival of mouse with pancreatic cancer, illustrating their therapeutic potentialities.

Garcia-Romero et al. [23] showed that all three types of EVs (Abs, MVs, and EXOs) secreted by human glioma cells contained gDNA sequences. Some sequences appeared in all EVs, whereas a few sequences appeared exclusively in one type of EVs. *IDH1*, harboring the most relevant mutation for human glioma diagnostic, was detected only in MVs and EXOs. Moreover, in vivo studies demonstrated that all types of tumor-derived EVs cross the intact blood–brain barrier and can be detected in the peripheral blood. In a small cohort of glioma patients, they demonstrated that the *IDH1^G395A^* mutation could be successfully detected in the peripheral blood EVs cargo as a minimally invasive method compared to liquid biopsy from cerebrospinal fluid. In 2019, Kahlert [24] wondered whether the exosomal gDNA, discovered only some years ago, might be a better choice as a cancer biomarker in liquid biopsy than the cfDNA discovered six decades before. After recapitulating the origin of both DNAs and their respective advantages and disadvantages, he concluded that both are currently complementary. Whereas cfDNA can be detected in healthy individuals and patients with nonmalignant or malignant disease, mutated cfDNA is more tumor-specific and enriched in smaller fragments between 90 and 150 bp and in the size range 250 to 320 bp, originating from cell death remnants insufficiently cleared by infiltrating phagocytes; therefore, cfDNA, with an easier amount of accessible DNA and higher copy numbers of some cancer-specific mutations, is more efficient for prognosis of late tumor stages. By contrast, exosomal gDNA can be found less fragmented (with a size range between 2.5–10 kb [10,11]) not only in exosomes, but in all EV types, more frequently in MVs and EXOs and sometimes only in some specific EXO subsets [11], with an apparent distribution depending on the tumor type. Although in a smaller amount, exosomal gDNA, spanning all the chromosomes, is sufficient to obtain the significant tumor-specific mutated DNA sequences by using the most recent PCR technologies. Thus, exosomal DNA might be a better potential biomarker for early cancer diagnosis than cfDNA. However, the clinical translation of exosomal DNA as a cancer biomarker is greatly hampered by the urgent need for finding a valuable substitute to the “gold standard” of differential ultracentrifugation for EVs extraction from human body fluids. The greatest promise for using the tumoral EV-specific gDNAs as an early cancer diagnosis biomarker might be to specifically extract tumor EVs from the whole circulating EV population by capture on “lab-on-chip” solutions, for example, by targeting some tumor-specific EV outer membrane proteins, such as glypican-1, followed by the use of the new PCR technologies for reaching the cancer-specific mutation(s) of interest.

To define EV component(s) as potential biomarker(s) for a given human cancer diagnosis by liquid biopsy, three steps are generally undertaken: in vitro studies with specific tumor cell lines, in vivo studies with murine tumor models, and preclinical studies on circulating tumor-derived EVs from a few patients’ plasma or serum. Whereas two-dimensional (2D) cell cultures are generally used as “gold standard” in vitro models, Thippabhotla et al. [25] intended to compare the EVs respectively released by an immortalized HeLa (2D) cell culture, issued from a cervical cancer patient, and a three-dimensional (3D) organoid culture, elaborated on peptide hydrogel with the same HeLa cells. They found that the EV secretion dynamics were significantly different for both culture types. Moreover, their respective EV-RNA and EV-DNA compositions were also quite different. The 3D-culture-derived EV-small RNA profile (<200 nt) showed a much higher similarity (about 96%) than the 2D culture-derived EVs to plasma EV-small RNA profile from two cervical cancer patients with one healthy control. In contrast with RNA, analysis of the cir-tEV-DNA sequencing data showed that culture or growth conditions do not affect the genomic DNA information carried by EV secretion. Therefore, at least for cervical cancer, 2D culture seems to remain a valuable in vitro tool for the search of human cir-tEV-gDNA cancer biomarker, whereas the 3D culture system may constitute a more useful in vitro model for the search of cir-tEV-RNA cancer biomarkers. Yokoi et al. [26] were the first to question the mechanisms of nuclear content loading to exosomes. Upon induction of genomic instability with genotoxic drugs, they identified a link between micronuclei (MN) formation and the generation of some specific exosomal loading with gDNA and other nuclear contents.

On the other hand, Lazaro-Ibanez et al. [27], using two human mast (HMC-1) and erythroleukemic (TF-1) cell lines, prepared, by ultracentrifugation, exosome-enriched small extracellular vesicles (sEVs). The amount of sEVs for TF-1 cells was over 2.5-fold more than that for HMC-1. By further using a high-resolution iodixanol density gradient on the two sEVs populations, the authors discriminated two novel heterogeneous subpopulations with different DNA content and topology. Each sEVs fraction was separated in nine 1 mL fractions (F1–F9) with measured densities from top to bottom. For both cell lines, the respective (F1 = F7) fractions were clustered in two low-density (LD) (F1–F3) and high-density (HD) (F4–F7) sEV subsets. The majority of the classical exosome-like sEVs were contained in the LD fractions. DNA was less abundant than RNA, and DNA was mainly present as ssDNA in the HD fractions for both cell types. The (HMC-1) HD fraction had a DNA-to-RNA ratio of 2.2/1, while the (TF-1) HD fraction was enriched in RNAs with a 1/2.9 DNA-to-RNA ratio. The LD fractions had the most prominent rRNA peaks and least DNA, while the HD fractions had most of the DNA cargo and small RNAs with no ribosomal rRNA peaks. DNA was predominantly localized on the outside or surface of sEVs, with only a small portion inside the vesicles. The entire human genome was represented both on the inside and outside of the sEVs. When sEVs were analyzed in bulk, whole-genome sequencing identified gDNA fragments of various lengths (from 500 to 10,000 bp), spanning both mitochondrial DNA and all chromosomes. These interesting and somewhat amazing observations have to be further explained, especially the cell mechanisms for the sEV specific loading before release and the curious DNA topology.

In 2019, Jeppesen et al. [28] questioned the heterogeneity of the exosome-enriched crude sEVs sample. From their in-depth studies published in *Cell*, the authors claimed the necessary reassessment of the “classical” exosome composition both with regard to their assumed biogenesis and to their widely admitted global composition. The most “iconoclast” assertion for the topic of the present review was that extracellular dsDNA was not associated with exosomes or any other types of sEVs. Reviewing the ongoing studies from 2020 might perhaps clarify this pending question concerning exosomal DNA, which is important for keeping the current assumed interest of EVs as a potential rich tumor DNA resource for early cancer diagnosis (cf. detailed discussion in part 5.). A Summary of these (2014–2019) studies about circulating tumor EVs-DNAs can be found in Table 2.

## 4. Preclinical Studies about the cirtEV-Derived gDNA as a Potential Cancer Biomarker (2020–2021)

In line with the prestigious, newly reassessed exosome description [28], Hoshino and 116 coauthors [29] brought, also in *Cell*, a more-medical insight by investigating the proteomic profile of potential new liquid biopsy cancer biomarkers in 426 human cancer and non cancer samples derived from various cells, tissues, and body fluids. However, instead of using two-pooled LD and HD density fractions of the crude sEVs [27], the authors categorized the crude sEVs into three prominent subpopulations: small exosomes (Exo-S 50–70 nm), large exosomes (Exo-L 90–120 nm), and exomeres (non vesicular (NV) particles <50 nm), collectively referred to as extracellular vesicles and particles (EVPs), with the aim of defining EVP protein signatures that distinguish cancer patients from healthy individuals. Exomeres were identified in 2018 as nanoparticles distinct from EVs by using asymmetric flow field-flow fractionation (AF-4) for EV analysis [30]. Among their in-depth studies [29], the authors analyzed 120 plasma-derived EVP proteomes from 77 cancer patients with 16 different cancer types and 43 healthy controls (HC). They highlighted the identification of EVP markers, characterized EVP markers in human tissues and plasma, and suggested that EVP proteins can be useful for cancer detection and determination of cancer type. For the present review focused on exosomal DNAs, it is noticeable that not only is the choice of the EV-transported components (proteins/RNAs/DNAs) as the best type of cancer biomarkers still widely questioned, but even the more appropriate nature of the circulating extracellular transporter (EVs and/or NV materials) is also becoming a matter of debate.

Although aware of the recent reassessment of the composition of EVs and the overturn of some previous findings [28,29], Teng and Fussenegger [31] kept the EV common classification in three main types (Exos, MVs, and ABs) for extensively reviewing the EV biogenesis, focusing mainly on exosomes and microvesicles. They detailed the current knowledge about the three distinct steps concerning exosomes biogenesis and release, initiated from the endosomal pathway, with further intracellular transport of the multivesicular bodies (MVBs) containing intraluminal vesicles, and fusion of some MVBs with the plasma membrane for exosomes release. Likewise, they detailed the mechanisms of biogenesis and release of microvesicles and discussed the current knowledge upon EV uptake and cell–cell communication, as well as upon the cargo sorting into EVs. Lastly, with all this accumulated knowledge, they concluded by recapitulating the many possible EV bioengineering methodologies for therapy improvements in the future.

Besides the increasing knowledge about EVs’ biogenesis and composition, some recent reviews were focused on potential EV-derived DNAs as liquid biopsy biomarkers applied on a few specific cancers. Thus, Kim et al. [32] were concerned with lung adenocarcinoma. After summarizing older liquid biopsy approaches to overcome the small tissue availability in lung cancer patients, they advocated for EVs as ideal carriers of cancer biomarkers. They recalled that, contrary to the passively released fragmented cfDNAs (about 200 bp), cirEV-DNAs consist of both large-sized ds-gDNAs (up to 10 kb) and fragmented mutated DNAs, giving an active image of both the viable and dying tumor cells. Moreover, a higher sensitivity can be achieved by using EV-DNAs obtained from bronchoalveolar lavage fluid (BALF) than those from blood. Compared with the short half-life (2–2.5 h) of cfDNAs, the membrane-protected EV-DNAs also have a high stability. In conclusion, cirEV-DNAs are expected to be more widely used in the future, when their current sophisticated isolation methods will become clinically adapted. By contrast, Sun et al. [33] claimed an improved detection of cell-free tumor DNAs (cf-tDNAs) in EVs-depleted plasma of cancer patients. It is to be stressed that exosomes were prepared either by mere precipitation using ExoQuick (System Biosciences, CA, USA) or fractionated by using five sequential centrifugations and ExoQuick instead of ultracentrifugation. However, preparing exosomes by a precipitation method might not be a guarantee for keeping the exosomal DNA cargo intact, and it is noticeable that, in this case, the exosomal fraction 5 was dominated by small (~160 bp) nucleosome-like DNAs [33]. It is also noticeable that an older research article [34], using two different methods for exosomes isolation, brought contradictory evidence that more than 90% of cfDNA in human blood plasma is localized in exosomes. However, agarose gel electrophoresis of DNA isolated from plasma exosomes showed two prominent bands, one high intensity and high molecular weight, and the other of low molecular weight (less than 200 bp in length). By RNase treatment, the first band turned out to be exosome copurified RNA, with a 5-fold higher amount than the exosomal dsDNA, corresponding to the second band. It would be worth performing some in vitro studies about the exosomal DNA yield and size as a function of the methods used for collecting the exosomes. Cambier et al. [35] aimed to identify circulating nucleic acid sequences associated with serum EVs as a step toward an osteosarcoma (OS) early detection assay. qPCR analysis of PEG-precipitated EVs revealed the over-representation of some repetitive element DNAs in OS patient versus control sera. Taken into account that, in these serum EVs the OS-associated repetitive element DNAs were sensitive to DNase I, they were not in a protected EV cargo. Moreover, the repetitive DNA elements were copurified with EVs in PEG precipitation and size exclusion chromatography (SEC), but not in CD81 or CD9 EV immunoaffinity capture. These observations were taken as supporting the recent exosome reassessment [28], claiming that exosomes do not contain DNA, or tightly associate with other non vesicular entities containing dsDNAs that are extruded from cancer cells. Ruhen et al. [36] aimed to use low-pass whole-genome sequencing to identify copy number variants (CNVs) in serial samples of both cf-tDNA and EV-DNA from plasma of a patient with metastatic breast cancer. Of the 52 CNVs identified in tDNA, 36 (69%) were detected in at least one cf-tDNA sample and 13 (25%) in at least one EV-DNA sample. Variants ranged in size from 0.3 to 106.5 Mb and were distributed randomly throughout the genome. Both kinds of noninvasive liquid biopsy depicted a CNV increase with disease progression, but this case study demonstrated that cf-tDNA, shed from apoptotic tumor cells, had a greater sensitivity for serial monitoring of breast cancer than EV-DNA actively secreted from viable neoplastic cells. Elzanowska et al. [37] summarized the biological and clinical aspects of EV-DNA and examined the current role of EV-DNA specifically in cancer. Overall, they emphasized that EV-DNA as a biomaterial for liquid biopsies is a new but definitely promising area of study, but its study in the clinical context is still quite open for further validation. Lee et al. [38] performed targeted NGS of DNA derived from bronchoalveolar lavage fluid (BALF-EV DNA) of 20 patients with *EGFR*-mutated non small cell lung cancer (NSCLC) and DNA from matched formalin-fixed paraffin-embedded (FFPE) tissue samples. EVs from BALF were heterogeneous (100–300 nm in size); EV-DNAs from the BALF existed in short and long sizes, but mostly in about 11 kb; and EVs contained DNAs from both vesicle surface and inside. The DNA yield from BALF-EVs was 100-times less than tissue DNA but had enough tumor-specific DNA for use in NGS analysis for the identification of actionable genetic alterations. This approach has a high potential clinical feasibility and utility. Kim et al. [39], also enrolling NSCLC patients after tyrosine kinase inhibitor therapy, compared different technological tools to detect *EGFR* mutations in 54 plasma samples and 13 pleural fluids. They demonstrated that combined tumor nucleic acid analysis (exoTNA+cfTNA) in the plasma and exoTNa in the pleural fluid allowed for the detection of target mutations more sensitively than that using cfDNA or total DNA alone. Amintas et al. [40], claiming that “dsDNA in EVs might be the latest most promising biomarker of tumor presence and complexity”, focused on the recent knowledge on the DNA inclusion in vesicles, the technical aspects of EV-DNA detection and quantification, and the use of EV-DNA as a clinical biomarker. They recapitulated the cell-free DNA cell sources by active or passive mechanisms (cf. their Figure 1) and summarized the tumor genome hallmarks reflected by EV-DNA, as well as the results of the main clinical studies assessing the performance of EV-DNA biomarkers (cf. their Table 1). Although suggesting EV-tDNA as an alternative to reach the promise of cftDNA, they concluded by enumerating the many challenging questions remaining to be solved before reaching this goal. Maire et al. [41] investigated whether the DNA in glioblastoma cell-derived EVs reflects genome-wide tumor methylation and mutational profiles and allows noninvasive tumor subtype classification. They found that DNA is present in the vast majority of EVs, with a major localization to the EV surface. Genome-wide methylation profiling identified with high accuracy in EV-DNA the methylation of the parental tumor-specific mutations and copy number variations (CNVs). Interestingly, the methylation profiling and CNV results were not affected by the EV isolation techniques. This showed that EV-DNA reflects the genome methylation, CNV, and mutational status of glioblastoma cells. Likewise, Baris et al. [42] compared epigenetic alterations in the target gene Enhancer of Zeste Homolog-2 (*EZH-2*) between plasma-derived exosomes and matched primary tumor tissues of 21 patients with aggressive diffuse large B cell lymphoma (DLBCL). They showed, for the first time, the presence of DNA in plasma exosomes of DLBCL patients and found that *CDKN2A* and *CDKN2B* were methylated in both plasma exosomes and primary tumor tissue samples. Compared to 21 healthy individuals, exosome concentration was approximately six-times higher in DLBCL patients, but the exosomal dsDNA content was extremely low compared to RNA contents. Zavridou et al. [43] were also the first to perform a direct comparison of gene expression and DNA methylation markers in CTCs and paired plasma-derived exosomes. This revealed a remarkable heterogeneity on gene expression and DNA methylation markers between EpCAM-positive CTCs and paired plasma-derived exosomes in metastatic castration-resistant prostate cancer (mCRPC) patients, with a significantly higher positivity in CTCs. Lastly, Hur and Lee [44] extensively reviewed the properties of extracellular vesicle-derived DNA for future clinical applications. They examined the biogenesis of DNA-containing EVs, their DNA methylation, and the use of next-generation sequencing (NGS). They questioned the use of EV-DNA as a biomarker in clinical settings, the modality of EV-DNA gene transfer, and its therapeutic potential. They hypothesized that DNA might exist inside an EV in a protected nucleosome or supercoiled form, which would enable the packaging of long dsDNA. Taking into account the nucleosome’s 11 nm size, long dsDNA would more likely be present in larger EVs. However, the presence and topology of DNA in extracellular EVs will continue to be controversial until the development of a method for isolating pure EV subsets. Nonetheless, the authors recalled that the (100 bp to 20 kbp) EV-dsDNA fragments can represent the entire genome and reflect the mutational status of tumor parental cells. Lastly, mentioning several recent liquid biopsy studies in different body fluids of EVs associated-dsDNA for cancer patients, they also expressed the strong interest of EV-DNA as a new potential cancer biomarker. A summary of the discussed preclinical studies (2020–2021), about the circulating tumor-derived EV- gDNA as a potential cancer biomarker, is given in Table 3.

## 5. Evolution of the Knowledge about the Composition of the Extracellular Environment (2019–2021)

During many years, the exosome concept was “the tree that hid the forest of EVs”. However, EVs “came on stage” about one decade ago and have been studied worldwide since 2012, reaching a huge, still-uncontrolled complexity in heterogeneity. An EV classification into three main categories as a function of their size and biogenesis, i.e., apoptotic bodies (ABs), microvesicles (MVs), and exosomes (EXOs), obtained a general long-lasting consensus until Jeppesen et al. [28] recently proposed a complete reassessment of exosome composition, with a new classification of low-density (LD) “exosome-like” small extracellular vesicles (sEVs), without any DNAs in their cargos, and a much more significant high-density (HD) non-EV extracellular mixed component associated with DNAs. Therefore, they used different cell lines, human plasma, and tissue for preparing sEVs samples by the commonly used differential centrifugations. Then, they further used a density discrimination by means of a discontinuous iodixanol gradient density. Being aware of the ultracentrifugation-induced aggregation artefacts, they kept, in parallel, parts of the 15,000× *g* filtered supernatants as precleared media. These media were submitted to direct immunoaffinity-capture (DIC) of exosomes by means of magnetic beads conjugated to exosomes-specific tetraspanins antibodies. The crude sEVs, their different density fractions, and the scarce directly captured CD63-, CD81-, or CD9-specific EVs were submitted to the same immunoblots. Different studies aimed to give an insight upon the proteins, RNA, and DNA composition of two-pooled low-density (LD) and high-density (HD) fractions of the crude sEVs samples. Surprisingly, many of the presumed components of exosomes were absent from the “classical” exosomes expressing CD63, CD81, and CD9. Many of the most abundant miRNAs were associated with extracellular nonvesicular (NV) fractions rather than with purified sEVs. Moreover, extracellular dsDNA was stressed as being not associated with exosomes or any other types of sEVs. An autophagy- and multivesicular endosome-related pathway was supposed to be the driver of extracellular DNA secretion instead of the exosome-dependent pathway. These assertions were sufficiently “iconoclast” to be seriously questioned before entering into the many details suggested for supporting the new exosome model. The results in [27] were indeed “interesting and amazing”, but when compared with those recalled in [28], both taken together were quite perturbing. The methods used for flotation, although with the same technology of discontinuous iodixanol gradient density, were not exactly the same (12–36% and 6–30% for the [28] gradients and 20–45% for the [27] one). Neither was on the same samples, and they used different means of sample deposit to the bottom of the centrifugation tube, i.e., 1 mL of crude sEVs suspension in PBS was mixed with iodixanol to a final 36% concentration [28] or mixed with 3 mL of a 60% iodixanol solution [27]. Moreover, the resulting increasing densities from top to bottom were differently measured, either with a refractometer on a mock identical gradient without sample [28] or directly on all the 1 mL collected fractions by absorbance at 340 nm [27]. The final results were indeed analogous for the LD fractions covering the “classical” exosomal sEVs. However, they were so different with regard to the HD fractions, corresponding either to a sum of many nonvesicular extracellular materials [28] or to another “non-classical” exosome subset [27], that it would be worth further questioning the properties of the discontinuous iodixanol gradient density method as a function of the chosen parameters on the same crude sEV sample. Although quite new and highly cited by further publications [29,31,33,35,36,37,40,41,44,45,46,47], the conclusions asserted by Jeppesen et al. [28] were only poorly confirmed [29,35]. Their claimed absence of exosomal DNA [28] did not appear to be quite convincing [33,37,40,41,44,45,46,47], especially when compared with the observations of Lazaro-Ibanez et al. [27]. These authors, also using a discontinuous iodixanol gradient density separation of crude sEVs, reached only two heterogeneous (LD and HD) subpopulations of sEVs and only a small discarded heavier fraction of non-EV material. Sun et al. [33], taking into account the suggestion that extracellular DNA may not be associated with exosomes, but copurifies with the sEV fraction during standard isolation protocols [28], elaborated a clinically feasible protocol to analyze the cirEVs influence on the whole plasma cf-tDNAs’ measurements. The authors selected nine small-cell lung cancer (SCLC) patients with a known relatively high cf-tDNA content; for each patient, they prepared, from a 1 mL blood sample, a platelet-poor conventional-plasma and, from another ml of the same blood sample, four pelleted fractions by successive light centrifugations, with replacement of the usual last ultracentrifugation by an ExoQuick exosome precipitation. Thus, the fractionated plasma corresponded, respectively, to “cells and larger debris” (fraction 1); crude “large microvesicles” (fraction 3); exosomes (fraction 5), which were characterized by transmission electron microscopy (TEM); nanoparticle tracking analysis (NTA); and by CD63/CD81 ratio using flow cytometry. The last supernatant (fraction 6) corresponded to the conventional plasma depleted of EVs. Then, the DNA yield and size distribution were compared in the whole plasma and in the different fractions for the nine (SCLC) patients. From 1 mL starting plasma, the average DNA yield was 5.3 ng in fraction 1, 1.73 ng in fraction 2, 0.99 ng in fraction 3, 0.68 ng in fraction 4, 4.17 ng in fraction 5, and 4.28 ng in fraction 6, and the average summed DNA yields in fractions 1, 5, and 6 accounted for 79.9% of the total DNA yields. Comparatively, whole plasma showed an average of 23.84% cftDNA in the same group of patients. The DNA size distribution was also measured in each DNA sample and showed a peak size of 7000–10,000 bp in fraction 1 and gradually reduced in fractions 2–3, while smaller fragments (about 160 bp) gradually increased from fractions 3 to 6. They also estimated cir-tDNA content in the different fractions and showed that the copy number variations (CNVs) were more detectable in fractions 3 (large EVs), 5 (exosomes), and 6 (EV-depleted plasma). Interestingly, the authors “were not able to remove any DNA copurified with exosomes”, as previously suggested [28], and therefore, they questioned the origin of cir-tDNA detected in fraction 5. Maire et al. [41] observed, in glioblastoma cell-derived EVs, that even after robust digestion of surface-associated DNA and any possibly contaminating free-floating DNA, they still detected DNA in 76.4% of the CD63/CD81-positive vesicles, strongly supporting the notion that EVs contained DNA inside. Some others tried reserved contradictory comments toward the suggested reassessment of exosome composition [28]. Thus, Elzanowska et al. [37] pointed out “the unreported amount of exosomes used in the study, as well as a limited cell lines included in the report”. For Hur et al. [44], the inconsistency about the presence or absence of exosomal DNA can be attributed to the preparation method and size of the isolated EVs. Zhou et al. [45] advocated exosomal DNA as possessing more abundant biological information and higher accuracy for prognosis prediction than cf-DNA in liquid biopsy. However, they recognized that it is unclear whether gDNA exists in exosomes in all mentioned studies with different DNA detection methods. Shen et al. [46] asserted that “too strict an exosome isolation strategy may result in the loss of DNA-containing vesicles”. Kalluri and Lebleu [47], summarizing the hallmarks of exosomes as being “a cell-to-cell transit system in the human body with pleiotropic functions”, mentioned the current controversy about exosomal DNA and gave a negative appreciation of ref. [28], which “did not specify the quantity of exosomes used in its analytical assays, leading to ambitious conclusions”.

However, the pioneering studies of Jeppesen et al. [28], stressing the importance of extracellular nonvesicular particles as DNA biomarkers, was highly comforted by the discovery of exomeres, using the new technology of asymmetric flow-field fractionation (AF-4) for identification of subsets of extracellular vesicles [30]. Furthermore, Zhang et al. demonstrated the exosome-like ability of exomeres to transfer functional cargoes [48]. Malkin and Bratman [49], focusing on the increasing huge heterogeneity of the extracellular medium, brought an outstanding review article about “the nomenclature of EVs and extracellular particles (EPs), the physical and structural characteristics of EV/EP DNA, the physiological roles of EV/EP DNA in health and disease and the emerging potential of EV/EP DNA as a molecular biomarker.” Interestingly, they extended the consensual long-lasting EV classification to nonvesicular EPs and modified the current nomenclature of extracellular components into large EVs (100 to >1000 nm), including apoptotic bodies (ABs), large oncosomes (LOs), microvesicles (MVs), originating from the plasma membrane; small EVs, including 50 to 130 nm exosomes (EXOs) of endosomal origin; and extracellular particles (<50 nm), including exomeres, with mean diameter of 35 nm, and chromatimers, both of yet unknown origin. Thus DNA, the overlooked component of EV/EPS is now becoming the central actor of many pending unanswered questions [49].

## 6. Challenging Questions to Solve before Clinical Use of cirEV-tDNAs and Technological State of the Art about EVs Isolation and Characterization

The assets of circulating EV-DNAs, as a new promising biomarker for cancer diagnosis and prognosis, have been convincingly demonstrated. However, the clinical transfer of the accumulated preclinical knowledge that began about one decade ago is highly hampered by some important challenging questions needing to be solved as a priority. The suppression of the main “bottlenecks”, both biological and medical, in the present knowledge about the extremely heterogeneous tumor-derived EVs/EPs populations, is highly dependent upon the future technological advances about their specific isolation and characterization. All the cells present in a human body, whether procaryotes or eukaryotes, are potentially equipped with the general cell property of releasing extracellular material, aimed either to remove no-longer-employed cell components or to send important epigenetic messengers into blood and/or into the many other minor subpopulation body fluids for modifying the fate of some specific recipient cells. Among this newly discovered “stellar” complexity of active extracellular material, it is not yet possible to precisely define the few tumor-specific subpopulations. At a smaller level of complexity, a given tumor cell population releases a quasicontinuum of EVs, with partly overlapping sizes and some common outer membrane protein markers. Therefore, the necessary classification of EV subsets without any specific biomarker is currently out of reach, which precludes further evidence for any of their specific biological functions. Moreover, the mechanisms used for specifically loading the multi components (proteins, lipids, nucleic acids, metabolites) into each EV cargo are almost completely unknown. This is also true for the EV-transported DNA, with some supplementary controversial questions about its topological localization inside the EV, outside on the EV membrane, or in both positions, and also on its size and nature as ssDNA, dsDNA, gDNA, or nucleosomes. The same questions will probably arise with the more recently discovered EPs, together with the one about the part played by the older known proteins such as Argonaute in the protected intercellular DNA transport. This detailed picture is aimed to show the huge problem of EV/EP heterogeneity, which has to be at least partly solved before efficiently facing the medical validation of a few promising EV-derived biomarkers for cancer diagnosis by well-standardized protocols for a given cancer, undertaken with important patient cohorts in different cancer centers worldwide.

Some recent technological reviews have been selected to give an insight into the current state of the art concerning EV isolation and characterization [50,51,52,53,54,55,56,57,58,59]. Valencia and Montuenga [50] focused on the biological properties of exosomes and especially on their heterogeneity, which is due to the association of five factors: the cell of EVs origin, the EVs size and number, their molecular composition, and their functionality transferred in recipient cells. Different combinations of these factors result in highly complex EV heterogeneity. Moreover, in an oncologic patient, tumor-derived exosomes are estimated to be no more than 10% of all the circulating exosomes. Nevertheless, the authors suggest that exosomal DNA might become the future liquid biopsy gold standard. However, to become a clinical reality, every single procedure (EV isolation and characterization and all analytical protocols) remains to be standardized for a valid comparison of the different EV-DNA biomarker studies. Saad et al. [51] detailed eight exosome-isolation methods and discussed the advantages and disadvantages associated with each method (cf. their Table 1). They also discussed the physical and chemical characterization and the detection techniques for exosomal samples.

Widely studied since 2012, exosomes/EVs, with their potential to develop new clinical approaches of modern medicine, are also progressively entering the medical field, especially in cancer, cardiovascular disease, and central nervous system defects [52]. Hirata et al. [53] summarized the assets of liquid biopsy as a distinctive approach to the diagnosis and prognosis of cancer. They strongly advocated liquid biopsy compared with the usual tissue biopsy and its drawbacks. Although mentioning exosomes, they only actualized the comparison between the two older liquid biopsy circulating biomarkers CTCs and cell-free tDNAs as cancer diagnostic and prognostic tools.

With regard to compared EV characterization between tumors and normal controls, Western blots and all the “omics” technologies, i.e., proteomic, transcriptomic, metabolomic, lipidomic, and genomic, gave, at each level, an interesting global insight of the tumor-induced modifications. Recently, Shaba et al. [54] reviewed the EV multiomics integrated approach and summarized the state of the art of EVs omic studies. The abundant information reached for each omic level has to be correctly deciphered, and this is even more necessary if the different omics levels interact together. One essential requisite for multiomics integration is, beyond the generation of different omic datasets from the same biological samples, the development of statistical and annotation tools, which is essential for the interpretation of data. Still, many issues are encountered in each step of EV multiomic analysis, starting from EV isolation to the data integration methods, suggesting that this field is at its early state and requires further improvements. However, considering the complex EVs as optimal targets for omic sciences, the authors predicted a future challenging milestone for a multiomic integrative approach, which might contribute to explore EV functions, their tissue-specific origin, and their potentiality. On the other hand, it is feasible that each cancer-related global EV description might be “blurring” minute but important EV subsets, specifically linked with the tumor processes. Therefore, the new recent analyses at the single extracellular vesicle level (SVA), summarized by Bordanaba-Florit et al. [55], seem to be a quite interesting complementary approach to unravel the heterogeneity of extracellular vesicles. The authors extensively described some of the current methods so far developed for single-vesicle analysis (SVA). They reviewed the assets of SVA methods on recent advances in the EV field of research. They also focused on prostate cancer (PCa) diagnostics, showing the important improvements brought by the SVA of EVs. Ultimately, they concluded that an entirely new cell-to-cell EV-mediated communication network will be founded by single-vesicle techniques. SVA is also bridging the “omic” studies, carried for deciphering the global EV properties and their further functional studies, to the clinical world, by participating in the elaboration of simpler and less time-consuming technologies for EV isolation, such as microfluidics. Recently, Mousavi et al. [56] extensively reviewed microfluidics for detection of exosomes and microRNAs in cancer. First introduced in the early 1990s, microfluidics manipulates microliter volumes in microchannels ranging in size only from 1 to 1000 µm. When compared with conventional studies, microfluidics platforms have many advantages, including enhanced reliability, sensitivity, accessibility, lower consumption of samples and reagents, reduced costs, quicker processing and response times, and the possibility of automated multiplexing. The authors summarized the microfluidic technologies used for exosome isolation and analysis and specifically applied for cancer studies. They also focused on microfluidic-based miRNA detection in human cancer. An increased interest has been shown in microfluidics use for biomarker discovery, but many challenges are yet to be faced, such as standardization and validation at a large scale, before any routine clinical application for cancer diagnosis. Recently, Campos-Silva et al. [57] described a simple immunoassay for extracellular vesicle liquid biopsy in microliters of unprocessed plasma. They demonstrated that many EVs in solution, being like stable colloidal suspensions, are therefore unable to interact with a stationary functionalized surface. A more efficient capture on antibody-coated surfaces was obtained by using flocculation methods with cationic polymers. This led to the optimization of a protocol allowing effective immunocapture of EVs in bead-assisted flow cytometry. Only a few microliters of plasma were necessary for easy detection of tumor markers without previous ultracentrifugation. This easily adaptable method has been validated using plasma from lung cancer patients, with detection of the epithelial cell marker EpCAM on EVs. This radically improves the efficiency of clinical EV detection in immunocapture assays, opening new possibilities for the validation of EV biomarkers with large cohorts of patients.

## 7. Conclusions

To gain a new step toward the clinical practice, it is mandatory to deeply investigate the still controversial nature and topology of the EV-associated DNA and the largely unknown EP-associated DNA. Moreover, microfluidics should focus on new technologies for discriminating circulating tumor EVs/EPs from the wide panel of the numerous other circulating EVs/EPs, blurring the tumoral message. As observed in this review focused on EV-DNA, the extracellular world is now becoming even more complex by the recent introduction of extracellular particles (EPs) [28,49], competing with EVs for assuming the many important intercellular messenger functions involved in human health and disease. It stresses the fundamental importance of deeply deciphering the extracellular environment composition and functions to complement the current knowledge slowly accumulated during two centuries about the cell machinery. As already mentioned [54], using a multiomics integrative approach at the single EV level [55] is probably the ultimate goal to elucidate the most challenging EVs/EPs complexity in the far future. Therefore, it is probably only the very beginning of a long-standing scientific query, highly dependent on many future technological advances to control the EV/EP-epigenetic extracellular heterogeneity governing their putative, specific intercellular functions. Besides overcoming these major challenges, it will be necessary to define a standardized protocol for analyzing each given promising liquid biopsy biomarker for a given cancer type. Finally, the essential large-scale intercenter clinical validation might bring the putative biomarker to the long-awaited clinical practice. To give an optimistic insight into the huge interest in these hard future steps, one can mention a recent editorial about exosomes in cancer therapy [58] and a hopeful commentary upon liquid biopsy of extracellular biomarkers for prostate cancer personalized treatment decision [59]. Moreover, a recently published new EV data base (EV-ADD), the first one to be concerned with EV-associated DNA in human liquid biopsy samples [60], corroborates the current potential interest of this long-neglected EV component, not only for early cancer diagnosis but also possibly in the future for prognosis and disease monitoring after treatment, and even for EV-mediated therapy and resistance to therapy.

## Figures and Tables

**Table 1:** Precursor studies on the DNA content of cell-derived EVs (2011–2016).

Cell Lines/Samples	Main Results	Reference
Three medulloblastoma cell lines.	MVs carry DNA which reflects the genetic status of the tumor with a significant amplification of the *c-Myc* oncogene; exoDNA is primarily single stranded.	[6]
Murine cardiomyocite muscle cell line.	MVs/exos containing DNA/RNA could transfer chromosomal DNA sequences to target fibroblasts.	[7]
Human VSMCs culture and plasma. K562s and human neutrophils.	EV-mediated transfer of gDNA to recipient cells: a novel mechanism for intercellular genetic influence. Transfer of BCL/ABL hybrid gene from K562s-EVs to normal human neutrophils.	[9]
Two pancreatic cancer cell lines. Serum from PDAC patients.	Exos contain >10kb fragments of ds-gDNA spanning all chromosomes. Specific KRAS and p53DNA mutations found in serum exosomes of PDAC patients.	[10]
Three cancer model cell lines: human myeloid leukemia; human colorectal carcinoma; and murine melanoma.	The majority of DNA associated with tumor exos is double stranded either externally (>8.5 kb), larger than internal ExoDNA, or extended to a broad panel of tumor cell lines; in murine melanoma, only a 10% sExo subset contained DNA; exo-dsDNA reflects the mutational status of parental cells.	[11]
Two (IEC) rat cell lines, (nontumorigenic (RAT-1) and tumoral (RAS-3).	The RAS-3 EVs contained dsDNA large fragments, covering the entire rat genome, including the transferable full-length H-RAS oncogene (3308 bp).	[12]
Human mast cells.	The EV-DNA released by human mast cells is usually associated with the outside of EVs.	[13]
Human BM-hMSCs−/+ transduction with a plant DNA	The cell-derived EVs also carry high molecular DNA not originating from dying cells, mainly associated to the outer EV membrane, and not organized in nucleosomes. Confirmation of the EV-mediated horizontal gene transfer.	[14]
Review	Summary of ds-gDNA in circulating exosomes.	[15]
Serums	Stability of EVs extracted from serums under different storage conditions.	[16]

**Table 2:** Further studies on circulating tumor EVs-DNAs (2014–2019).

Samples/Aims	Main Results	Reference
Three prostate cancer (PCa) cell lines. Plasma of human (PCa) patients (n = 4).	Different gDNA fragments in the subpopulations of EVs (Abs, MVs, and EXOs). EV-gDNA could harbor specific gDNA mutations of the parent cells. Plasma EVs also carry double-stranded gDNA with no differences in MVs/EXOs.	[17]
Glioblastoma, PC3 prostate cancer, or U87 cancer cell lines. Plasma of a PCa mouse model; human plasma of mCRPC patients (n = 40).	Large EVs (oncosomes) contain most of the circulating chromatinized DNA (up to 2 Mb). L-EVs from human mCRPC patients contained large-sized dsDNA, covering the entire tumor genome, with reported cancer-specific (*MYC/PTEN*) genomic alterations.	[18]
Whole blood samples of pancreatic cancer (PDAC) patients (n = 127) and controls.	*KRAS* mutations were more detectable in exoDNA than in cell-free DNA, but mutant KRAS was also detected in a substantial minority of healthy samples.	[19]
Serum from patients with (PDAC) pancreatic cancer or pancreatic disease and from healthy individuals.	The minimal exosomal DNA used for digital PCR analyses was 0.663 ng. Potential clinical utility of circulating exosomal DNA for identification of *KRAS^G12D^* and *TP53^R273H^* mutations in patients with pancreas-associated pathologies.	[20]
Engineered exosomes from fibroblasts-like mesenchymal cells (iEXosomes).	Compared to liposomes, iExosomes facilitate therapeutic targeting of oncogenic KRAS in pancreatic cancer.	[21]
Bioreactor-based generation of clinical-grade iExosomes.	Large-scale production of clinical-grade iExosomes for targeting KRAS in pancreatic cancer.	[22]
Xenotransplant mouse model of human glioma-cancer stem cells featuring an intact blood–brain barrier (BBB).	The three types of glioma-derived EVs (ABs, MVs, and EXOs) contained gDNA sequences. Some sequences appeared in all EVs, whereas a few sequences appeared exclusively in one type of EVs. All tumor-derived EVs cross the intact BBB and can be detected in the peripheral blood.	[23]
Comparison of circulating cfDNA and EV-DNA, their origins, and their respective advantages and disadvantages for cancer diagnostic.	Mutated cfDNA, more tumor-specific and enriched in smaller fragments, is more efficient for prognosis of late tumor stages. Exosomal gDNA (between 2.5–10 kb) might be a better potential biomarker for early cancer diagnosis.	[24]
An immortalized HeLa cervical cancer (2D) cell culture and a three-dimensional (3D) organoid culture.	The EV secretion dynamics were significantly different for both culture types: 2D culture remains a valuable tool for the search of human cir-tEV-gDNA cancer biomarker, whereas the 3D culture seems more useful for searching cir-tEV-RNA.	[25]
Mechanisms of nuclear content loading to exosomes.	A link between micronuclei (MN) formation and the generation of some specific exosomal loading with gDNA was identified by inducing genomic instability.	[26]
Human mast (HMC-1) cell line and (TF-1) erythroleukemic cell line.	Exosome-enriched small extracellular vesicles (sEVs) were discriminated by a high resolution iodixanol density gradient into two novel heterogeneous EV subpopulations of low density (LD) and high density (HD) with different RNA/DNA EV cargoes.DNA was predominantly localized on the outside or surface of sEVs.	[27]
Human colon (DKO1) and glioblastoma (Gli36) cell lines; normal primary kidney epithelial cells and human plasma.	Necessary reassessment of the “classical” exosome composition and biogenesis: extracellular dsDNA is not associated with exosomes or any other types of small EVs, but with extracellular particles (EPs).	[28]

**Table 3:** Preclinical studies about the circulating tumor-derived EV-gDNA as a potential cancer biomarker (2020–2021).

Aims/Samples	Main Results	Reference
Proteomic profile of potential cancer biomarkers in 426 human cancer and noncancer samples derived from various cells, tissues, and body fluids.	Crude sEVs categorized into (EVPs) three subpopulations: small exosomes (Exo-S 50–70 nm), large exosomes (Exo-L 90–120 nm), and exomeres (non vesicular (NV) particles <50 nm). Analysis of 120 plasma-derived EVP proteomes from 77 cancer patients with 16 different cancer types and 43 healthy controls (HC) suggested that EVP proteins can be useful for cancer detection and determinization of cancer type.	[29]
Extensive review on the EV biogenesis, focusing mainly on EXOs and MVs.	Discussion about the current knowledge upon EV-uptake and cell–cell communication, as well as upon the cargo sorting into EVs. Possible EV bioengineering methodologies for therapy improvements.	[31]
Comparison of EV-mediated liquid biopsy with older liquid biopsies for lung adenocarcinoma diagnosis.	EVs are advocated for as ideal carriers of cancer biomarkers. Contrary to the passively released fragmented cfDNAs (about 200 bp), cEV DNAs consist of both large-sized ds-gDNAs (up to 10 kb) and fragmented, mutated DNAs. The membrane-protected EV-DNAs also have a high stability A higher sensitivity can be achieved by using EV-DNAs obtained from bronchoalveolar lavage fluid (BALF) than those from blood.	[32]
Nine small-cell lung cancer (SCLC) patients and twenty-two (SCLC) patients with known tumor EGFR mutation.	Platelet-poor plasma was fractionated by five sequential centrifugations and ExoQuick for preparing the exosomal fraction 5, which was then dominated by small (~160 bp) nucleosome-like DNAs.Improved detection of cell-free tumor DNAs (cf-tDNAs) is claimed in EV-depleted plasma (fraction 6), and higher mutation detection rates (14/22) are observed than in whole plasma (10/22).	[33]
Blood samples from healthy human donors.	This older study contradicts the previous one by showing the association of dsDNA inside the plasma exosomes and stating that “more than 93% of amplifiable cfDNA in plasma is located in plasma exosomes”.	[34]
Human osteosarcoma (OS) serum samples.	Copurification of OS-associated repetitive element DNAs with EVs in size exclusion chromatography but not in exosome immunoaffinity capture. Repetitive element DNAs showed a high sensitivity and specificity for sera of patients with an OS diagnosis but were not tightly bound to CD9+ or CD81+ exosomes, supporting that exosomes either do not contain DNA or are tightly associated with particles with DNA.	[35]
Comparison of cf-tDNA and EV-DNA in serial plasma samples of a metastatic breast cancer patient.	Of the 52 copy number variants (CNVs) (from 0.3 to 106.5 Mb) in tDNA, 36 were detected in at least one cf-tDNA and 13 in one EV-DNA sample and were distributed randomly throughout the genome. cf-tDNA, shed from apoptotic tumor cells, had a greater sensitivity for serial monitoring of breast cancer than EV-DNA actively secreted from viable neoplastic cells.	[36]
Summary of the biological and clinical aspects of EV-DNA and role of EV-DNA in cancer.	EV-DNA as a biomarker for liquid biopsy is a new but definitely promising area of study, but its study in the clinical context is still quite open for further validation.	[37]
Bronchoalveolar lavage fluid (BALF) of 20 (NSCLC) patients with *EGFR*-mutations and matched fixed-tissue samples.	Heterogeneous (100–300 nm) EVs from BALF contained mostly ~11kb DNAs from both vesicle surface and inside. The DNA yield from BALF-EVs was 100 times less than tissue DNA but had enough tumor-specific DNA for the identification of actionable genetic alterations with a high potential clinical utility.	[38]
54 plasma samples and 13 pleural fluids of (NSCLC) patients after tyrosine kinase inhibitor therapy.	By comparison of different technological tools to detect *EGFR* mutations, combined tumor nucleic acid analysis (exoTNA+cfTNA) in the plasma and exoTNa in the pleural fluid allowed for the detection of target *EGFR* mutations more sensitively than using cfDNA or total DNA alone.	[39]
Focus on the DNA inclusion in EVs, the techniques of EV-DNA detection and quantification, and the clinical use of EV-DNA.	Recapitulation of the cell-free DNA cell sources by active or passive mechanisms and summary of the tumor genome hallmarks reflected by EV-DNA as well as the results of the main clinical studies assessing the performance of EV-DNA biomarkers.Enumeration of the many challenging questions remaining to be solved before reaching the clinics.	[40]
Cell lines and glioblastoma stem-like (GS) cell cultures.Human glioma patients’ tissue and nontumoral tissue.	The vast majority of EVs carry DNA, which localizes more to the EV surface than inside EVs. Proof of principle that glioblastoma-derived EV-DNA reflects the genome-wide methylation, CNVs, and mutational status of glioblastoma cells with high accuracy and enables their molecular classification.	[41]
Plasma and matched primary tumor tissues of 21 patients with aggressive diffuse large B cell lymphoma (DLBCL).	First study to show the presence of DNA in plasma exosomes of DLBCL patients.*CDKN2A* and *CDKN2B* were methylated in both plasma exosomes and primary tumor tissue samples.Compared to 21 healthy individuals, exosome concentration was approximately 6 times higher in DLBCL patients, but the exosomal dsDNA content was extremely low compared to RNA contents.	[42]
First direct comparison on gene expression and DNA methylation markers in CTCs and paired plasma-derived exosomes.	Remarkable heterogeneity on gene expression and DNA methylation markers between EpCAM-positive CTCs and paired plasma-derived exosomes in metastatic castration-resistant prostate cancer (mCRPC) patients with significantly higher positivity in CTCs.	[43]
Extensive review of the characteristics and clinical applications of extracellular vesicle-derived DNA.	The presence of DNA in excreted exosomes will continue to be controversial until the development of a method for isolating pure exosomes or microvesicles. Nonetheless, the size of dsDNA found in EVs (from ~100 bp to ~20 kbp) can represent the entire genome and reflects the mutational status of tumor parental cells. With DNA extracted from all categories of EVs, EV-DNA is the latest and most promising biomarker for identifying tumor presence and complexity.	[44]
